# Prevalence and assemblage of *Giardia duodenalis* in a case-control study of children under 5 years from Jimma, Southwest Ethiopia

**DOI:** 10.1007/s00436-023-08029-5

**Published:** 2023-12-13

**Authors:** Yonas Alemu, Alemseged Abdissa, Zeleke Mekonnen, Bizuwarek Sharew, Øystein H. Johansen, Ola Bjørang, Nina Langeland, Kurt Hanevik, Sabrina J. Moyo, the CRYPTO-POC study group

**Affiliations:** 1 School of Medical Laboratory Sciences, Jimma University, Jimma, Ethiopia; 2 Department of Microbiology, Immunology and Parasitology, Addis Ababa University, Addis Ababa, Ethiopia; 3 Armauer Hansen Research Institute, Addis Ababa, Ethiopia; 4 Department of Clinical Science, University of Bergen, Bergen, Norway; 5 Department of Microbiology, Vestfold Hospital Trust, Tønsberg, Norway; 6 National Center for Tropical Infectious Diseases, Department of Medicine, Haukeland University Hospital, Bergen, Norway; 7 Department of Tropical Disease Biology, Liverpool School of Tropical Medicine, Liverpool, United Kingdom

**Keywords:** Prevalence, *Giardia duodenalis*, Assemblage B, Real-time PCR, Ethiopia

## Abstract

*Giardia duodenalis* is a common pathogenic intestinal protozoan parasite with high prevalence in developing countries, especially among children. The distribution of giardia assemblages among humans and their clinical relevance remains controversial. This study aimed to determine the prevalence and assemblage of *Giardia* among children under 5 years of age in Jimma, Southwest Ethiopia. Employing a case-control design, 606 children presenting with diarrhea at Jimma university medical center and Serbo Health Center were enrolled from December 2016 to July 2018 along with 617 matched controls without diarrhea. *Giardia* was detected and typed using real-time PCR. Univariate and multivariate regression analysis was performed. The total prevalence of *Giardia* was 41% (501/1223) and did not differ significantly between cases and controls (40% vs 42%). Prevalence increased by age, with the highest prevalence seen in children aged ≥ 25 months. Children without diarrhea with a history of diarrhea during the last month were more likely to be *Giardia* positive compared to children with no history diarrhea (OR 1.8 and 95%CI; 1.1–2.9). Regardless of current diarrhea symptoms, assemblage B predominated with 89%, followed by assemblage A (8%) and mixed infection assemblage A and B (3%). We report a high prevalence of *Giardia* by PCR detection in Jimma, Ethiopia, with assemblage B being predominant. There was a similar distribution of *Giardia* assemblages between children with and without diarrhea. Increasing age was a risk factor for *Giardia* infection. Community-based prevention and control strategies need to be employed to decrease the risk of giardia infection.

## Introduction

*Giardia duodenalis* (syn. *Giardia lamblia* and *Giardia intestinalis*) causes *Giardia* infection in humans and many mammals. It is transmitted through the oral-fecal route following direct or indirect contact with the infectious cyst stages, including human-to-human, zoonotic, waterborne, and foodborne transmission (Einarsson et al. 2016; Feng and Xiao 2011).

The spectrum of clinical symptoms that occur in infected individuals may range from asymptomatic to acute or chronic diarrheal disease. When present, the clinical signs may include diarrhea, nausea, weight loss, bloating, and abdominal pain (Adam 2021). Furthermore, chronic infection can result in stunting and reduced psychomotor development (Rogawski et al. 2017; Kabir et al. 2022). Giardiasis can also cause severe malabsorption resulting in malabsorption of fat, proteins, folic acid, vitamin A, and vitamin B12 (Kabir et al. 2022; Keselman et al. 2016; Al-Mekhlafi et al. 2010).

Giardiasis is especially common in areas with poor sanitation and no or insufficient water treatment facilities (Leder and Weller 2020). The prevalence of giardiasis ranges from 3 to 7% in developed countries and 20 to 30% in developing countries (Leung et al. 2019; Mahdavi et al. 2022) and even more than 30% in Ethiopia (de Lucio et al. 2016; Gelanew et al. 2007). The variation in prevalence might be attributed to factors such as the geographical area, urban or rural settings, age group composition, hygienic standards, and the socio-economic conditions of the study subjects.

Molecular studies reveal eight distinct genetic assemblages of *Giardia duodenalis*, i.e., assemblages A to H. Two of the assemblages, A and B, are widely known to infect humans and other mammals (Heyworth 2016). The remaining six assemblages (C to H) are host-specific and infect animals, even though assemblages C (Soliman et al. 2011), E (Fantinatti et al. 2016), and F (Gelanew et al. 2007; Pipikova et al. 2020) have been reported from human isolates. There is extensive geographical variation in *G. duodenalis* assemblages within countries and between different countries and continents (Hijjawi et al. 2022). For example within Ethiopia, some reported higher rate of assemblage B (Tigabu et al. 2010; Wegayehu et al. 2013; Birrie and Erko 2017) while other studies reported higher prevalence of assemblage A (Gelanew et al. 2007; Hajare et al. 2022) using different types of PCR techniques targeting glutamate dehydrogenase, beta-giardin, and/or triosephosphate isomerase genes or sequencing.

There are discrepancies in the reported *Giardia* prevalence within Ethiopia. Most available reports on prevalence of *G. duodenalis* infection in Ethiopia have used conventional microscopy for detection of *Giardia*. These studies in North Shoa, Benishangul Gumuz, Sidama, Lege Dini, Eastern Ethiopia, and Jimma town reported prevalence ranging from 5 to 35% (Ayalew et al. 2008; Belete et al. 2021; Beyene and Tasew 2014; Damitie et al. 2018; Flecha et al. 2015; Kifleyohannes et al. 2022; Mengistu et al. 2007; Wegayehu et al. 2016). Some studies used PCR for detection of *Giardia* and found prevalence ranging from 11 to 55% in Southern Oromia, central Ethiopia, Southern Ethiopia, Tigrai North Ethiopia, and in Northwest Amhara regions (de Lucio et al. 2016; Tigabu et al. 2010; Wegayehu et al. 2013; Birrie and Erko 2017; Hajare et al. 2022).

Studies on the molecular epidemiology of *G. duodenalis* from different parts of the world help to clarify possible relationships among genetic diversity of the parasite, clinical presentation, and environmental transmission dynamics (Feng and Xiao 2011; Cacciò and Sprong 2011). So far, there are no studies using PCR to detect and characterize *Giardia* in the larger Jimma area in Ethiopia. Therefore, using a case-control design, we aimed to determine the prevalence, molecular epidemiology, and risk factors of *Giardia* infection among under 5-year-old children in Jimma, Southwest Ethiopia.

## Methods and materials

### Study area

The study was conducted in Jimma town (Jimma University Medical Centre, JUMC), located 346 km to the southwest of the capital Addis Ababa, and Kersa woreda (Serbo health center) located 18 km to the east of Jimma. Jimma town has a total population of 120,960 and is the administrative center for 21 surrounding districts. Jimma is largely coffee producing agrarian community.

### Study design and period

This study was part of a larger prospective case-control study, which investigated diagnostic accuracy of LED-AP for cryptosporidiosis (Johansen et al. 2021), conducted from December 2016 to July 2018.

### Study participant

All children under 5 years of age presenting with diarrhea at JUMC and Serbo Health Center were included after obtaining parents’ or guardians’ written informed consent. The study enrolled children younger than 5 years who presented with diarrhea (three or more loose stools within the previous 24 h), or dysentery (at least one loose stool with stains of blood within the previous 24 h). The study included cases with prolonged (7–13 days) and persistent (≥14 days) diarrhea. Community controls without diarrhea in the preceding 48 h were enrolled concurrently by weekly recruitment plans using frequency matching to cases by sex, age stratum, and geographical location of households.

### Data collection and laboratory procedures

Demographic and clinical data were collected by study nurses using standardized case report forms by interview and from hospital records. All parents/caretakers of the participants were instructed and asked to provide a stool sample of their children with a screw-cap plastic container. Aliquots of stool samples were stored at −80°C until shipment to Vestfold Hospital Trust, Norway, for further processing. During the time the study was conducted a wet microscopy was performed as soon as the stool sample reached the laboratory. A result slip with the wet microscopy findings was immediately brought back to the treating health care worker (doctor or nurse). The presence or absence of *Giardia* cysts or trophozoites were reported on this result slip. The decision to treat was left with the treating clinical team who followed local standard treatment regimen.

Total nucleic acid extraction was performed on aliquots of stool samples. Briefly, a thawed aliquot of stool was added to (pre-made) 500 μl S.T.A.R. buffer (Roche) + 500 μl BLB (MagNA Pure bacterial lysis buffer, Roche) and vortexed, and the stool-buffer suspension was kept frozen. Stool-buffer suspension was then used for nucleic acid extraction with MagNA Pure 96 instrument, MagNA Pure 96 DNA, and Viral NA Large Volume Kit, and eluted in 100 μl. *Giardia* was detected using real-time qPCR run on a Light Cycler 480II, Roche, including primers and probes for the internal control (DNA process control kit, Roche) (Johansen et al. 2021).

All *G. duodenalis* positive samples were further analyzed using an assemblage-specific real-time PCR assay (Van Lith et al. 2015). Primers targeting assemblage-specific genes: Translation Initiation Factor (locus GL50803_39587) and Cathepsin L precursor (locus GL50581_3714) were used as markers for assemblages A and B, respectively using a Light Cycler® 480 instrument. Each real-time PCR reaction consisted of 2×LightCycler 480 SYBR Green I Master mix, 3 pmoles of each primer, 2 μl of genomic DNA, and sterile water up to a final volume of 20 μl. Experimental conditions consisted of 5 min incubation at 95 °C, followed by 45 cycles of denaturation at 95 °C for 12 s, annealing at 55 °C for 12 s, and extension at 72 °C for 12 s. Fluorescence data were collected as a single acquisition at the end of each cycle. The melting curve analysis was performed at the end of each reaction and consisted of 95 °C for 5 s, 60 °C for 1 min, and heating to 97 °C with continuous acquisitions. Negative controls (water instead of DNA) as well as DNA from *G. duodenalis* assemblage A WB Clone C6 ATCC strain and an assemblage B cyst isolate, verified by tpi sequencing were included in each run. Cycle threshold (CT) values and melting curve values were recorded.

### Statistical analysis

The statistical analysis was carried out using the SPSS® statistics program, version 20. Univariate and multivariate regression models were used to assess possible risk factors for acquiring *Giardia*. Results were interpreted using odds ratios, 95% confidence intervals, and significance levels. For analysis of acute malnutrition, children < 6 months, severe acute malnutrition (SAM) was defined as weight for-height *z*-score (WHZ) ≤ −3 of the WHO standard curves (WHO Multicentre Growth Reference Study Group 2006) and/or presence of bilateral edema involving at least the feet. Moderate acute malnutrition (MAM) was defined as a WHZ ≤ −2 and > −3 with no edema. Mid-upper arm circumference (MUAC) was used instead of WHZ for 6- to 59-month-olds, as it was difficult to bring height measurement boards to community-control home visits (usually done by motorcycle), and because MUAC is less susceptible to dehydration than weight (Modi et al. 2015; Mwangome et al. 2011); SAM was defined as MUAC ≤ 115 mm and/or presence of bilateral edema involving at least the feet, and MAM was defined a MUAC >115 mm and ≤ 125 mm with no edema. During analysis Acute malnutrition combined SAM and MAM.

## Results

### The study population

The study included 1223 children of which 674 (55%) were males and 549 (45%) were females. Out of 1223 children, 58% and 42% were from Serbo and Jimma study sites respectively. The most common age category was 7–12 months (33%), followed by the 13–24 months (32%). Using anthropometric measurements most children (88%) had no acute malnutrition, 51% kept animals in the house and 98% of children with diarrhea had acute diarrhea lasting less than 14 days (Table 1).

**Table 1:** Distribution of the study population at JUMC and Serbo HC (*N=*1223)

Demographic/clinical characteristics	Number (%)
Sex	
Male	674 (55.1)
Female	549 (44.9)
Age in months	
0–6	145 (11.9)
7–12	403 (33.0)
13–24	386 (31.6)
≥25	289 (23.6)
Study site	
Jimma	513 (41.9)
Serbo	710 (58.1)
Nutritional status (*N=*1218) ^a^	
No acute malnutrition	1074 (88.2)
Acute malnutrition	144 (11.8)
Animal keeping in the house (*N=*1206)^b^	
Yes	616 (51.1)
No	590 (48.9)
History of diarrhea in the last 1-month (*N=*1218)^c^	
Yes	190 (15.6)
No	1028 (84.4)
Type of diarrhea (*N=*605)^d^	
Acute (< 14 days)	593 (98.0)
Persistent (> 14 days)	12 (2.0)
History of vomiting (*N=*605)^d^	
Yes	343 (56.7)
No	262 (43.3)

^a^5 missing values; ^b^17 missing value; ^c^5 missing values; ^d^1 missing value

### Prevalence and quantity of *G. duodenalis* among the cases and controls study participants

Of the 1223 study participants, 606 and 617 were cases and controls, respectively. The prevalence of *G. duodenalis* was 40% (240/606) in cases and 42% (261/617) in controls. The prevalence of *Giardia* in participants of this study was not significantly different among cases and controls OR 1.1, 95% CI (0.9–1.2). Because history of diarrhea in the last one month was a risk for detecting *G. duodenalis* in stool, we did sensitivity analysis by excluding children with history of diarrhea in the past month. The prevalence of *Giardia* was quite similar in cases and controls 41%, 212/522 vs. 38%, 193/506 OR 1.12, 95% CI (0.9–1.4). We further assessed the quantity of *G. duodenalis* in the samples based on the Cq values obtained in qPCR using non-parametric test. We found a higher quantity of *Giardia* among children without diarrhea (mean Cq value 26.6) compared to children with diarrhea (mean Cq value 27.5) (*P*=0.006).

### Association between *Giardia* and different demographic and clinical characteristics

Tables 2 and 3 show the association between *G. duodenalis* and different demographic and clinical characteristics in univariate and multivariate analysis, respectively. The prevalence of *G. duodenalis* in study participants was increasing with age, the highest prevalence was seen among children ≥ 25 months in both cases and controls, OR 23.75, 95% CI (10.31–54.73) *P* = <0.001. Children without diarrhea, with a history of diarrhea in the past month had increased risk of having a *Giardia* positive stool sample (OR 1.8; 95%CI 1.1–2.9). Other demographic or clinical characteristics such as sex, nutritional status, domestic animals in the house, type of diarrhea, and vomiting were not associated with *Giardia* infection.

**Table 2:** Association between *Giardia* and demographic/clinical characteristics (*N=*1223) (univariate analysis)

Characteristic	Cases				Controls			
	*N*	*Giardia* +ve	*Giardia −*ve	OR (95%CI)	*N*	*Giardia +*ve	*Giardia −*ve	OR (95%CI)
		*n* (%)	*n* (%)			*n* (%)	*n* (%)	
Sex								
Male	361	144 (39.9)	217 (60.1)	1.03(0.74–1.44)	313	133 (42.5)	180 (57.5)	1.02 (0.74–1.39)
Female	245	96 (39.2)	149 (60.8)	1	304	128 (42.1)	176 (57.9)	1
Age in months								
0–6	86	6 (7.0)	80 (93.0)	1	59	6 (10.2)	53 (89.8)	1
7–12	214	58 (27.1)	156 (72.9)	**4.9 (2.05**–**11.98)**	187	42 (22.5)	145 (77.5)	**2.6 (1.03**–**6.36)**
13–24	177	90 (50.8)	87 (49.2)	**13.8 (5.72**–**33.27)**	209	116 (55.9)	93 (44.5)	**11.0 (4.54**–**26.76)**
≥25	128	86 (67.2)	42 (32.8)	**27.3 (11.01**–**67.69)**	161	97 (44.4)	64 (39.8)	**13.4 (5.44**–**32.97)**
Study site								
Jimma	251	85 (33.9)	166 (66.1)	1	262	110 (42.0)	152 (58.0)	1
Serbo	355	155 (43.7)	200 (56.3)	**1.5(1.08**–**2.11)**	355	151 (42.5)	204 (57.5)	1.0 (0.74–1.41)
Nutritional status (*N=*1218)								
Acute malnutrition	115	46 (40.0)	69 (60.0)	1.0 (0.67–1.54)	29	9 (31.0)	20 (69.0)	0.6 (0.27–1.35)
No acute malnutrition	490	194 (39.6)	296 (60.4)	1	584	249 (42.6)	335 (57.4)	1
Diarrhea in last month (*N=*1218)								
Yes	96	45 (46.9)	51 (53.1)	1.4 (0.92–2.22)	94	49 (52.1)	45 (47.9)	**1.59 (1.03**–**2.47)**
No	506	193 (38.1)	313 (61.9)	1	522	212 (40.6)	310 (59.4)	1
Keeping domestic animals (*N=*1206)								
Yes	300	117 (39.0)	183 (61.0)	0.9 (0.69–1.34)	316	128 (40.5)	188 (59.5)	0.9 (0.63–1.99)
No	294	117 (39.8)	177 (60.2)	1	296	130 (43.9)	166 (56.1)	1
Type of diarrhea (*N=* 605)								
Acute (<14 days)	593	237 (40.0)	356 (60.0)	1	NA	NA	NA	NA
Persistent (>14 days)	12	3 (25.0)	9 (75.0)	0.5 (0.13–1.86)	NA	NA	NA	NA
Vomiting (*N=*605)								
Yes	343	132 (38.5)	211 (61.5)	0.9 (0.64–1.24)	NA	NA	NA	NA
No	262	108 (41.2)		1	NA	NA	NA	NA

*NA*, not applicable for that analysis, bolded values are Confidence intervals that do not overlap the null value of odds ratio = 1

**Table 3:** Association between *Giardia* and demographic/clinical characteristics (*N=*1223) (multivariate analysis)

Characteristic	Cases				Controls			
	*N*	*Giardia* +ve	*Giardia* -ve	OR (95%CI)	*N*	*Giardia +ve*	*Giardia −*ve	OR (95%CI)
		*n* (%)	*n* (%)			*n* (%)	*n* (%)	
Sex								
Male	361	144 (39.9)	217 (60.1)	1.06 (0.73–1.54)	313	133 (42.5)	180 (57.5)	0.9 (0.69–1.41)
Female	245	96 (39.2)	149 (60.8)	1	304	128 (42.1)	176 (57.9)	1
Age in months								
0–6	86	6 (7.0)	80 (93.0)	1	59	6 (10.2)	53 (89.8)	1
7–12	214	58 (27.1)	156 (72.9)	**4.7 (1.92**–**11.51)**	187	42 (22.5)	145 (77.5)	**2.4 (1.0**–**5.9)**
13–24	177	90 (50.8)	87 (49.2)	**13.8 (5.67**–**33.68)**	209	116 (55.9)	93 (44.5)	**11.8 (4.72**–**29.65)**
≥25	128	86 (67.2)	42 (32.8)	**27.6 (11.01**–**69.23)**	161	97 (44.4)	64 (39.8)	**15.5 (6.08**–**39.52)**
Study site								
Jimma	251	85 (33.9)	166 (66.1)	1	262	110 (42.0)	152 (58.0)	1
Serbo	355	155 (43.7)	200 (56.3)	**1.8 (1.15**–**2.81)**	355	151 (42.5)	204 (57.5)	**1.6 (1.05**–**2.41)**
Nutritional status (*N=*1218)								
No acute malnutrition	115	46 (40.0)	69 (60.0)	1	29	9 (31.0)	20 (69.0)	1
Acute malnutrition	490	194 (39.6)	296 (60.4)	1.1 (0.70–1.85)	584	249 (42.6)	335 (57.4)	1.7 (0.67–4.19)
Diarrhea in last 1 month (*N=*1218)								
Yes	96	45 (46.9)	51 (53.1)	1.5 (0.93–2.51)	94	49 (52.1)	45 (47.9)	**1.6 (1.07**–**2.60)**
No	506	193 (38.1)	313 (61.9)	1	522	212 (40.6)	310 (59.4)	1
Keeping domestic animals (*N=*1206)								
Yes	300	117 (39.0)	183 (61.0)	0.8 (0.54–1.27)	316	128 (40.5)	188 (59.5)	0.8 (0.5–1.12)
No	294	117 (39.8)	177 (60.2)	1	296	130 (43.9)	166 (56.1)	1
Type of diarrhea (*N=* 605)								
Acute (<14 days)	593	237 (40.0)	356 (60.0)	1	NA	NA	NA	NA
Persistent (>14 days)	12	3 (25.0)	9 (75.0)	0.3 (0.07–1.85)	NA	NA	NA	NA
Vomiting (*N=*605)								
Yes	343	132 (38.5)	211 (61.5)	1.0 (0.69–1.50)	NA	NA	NA	NA
No	262	108 (41.2)	154 (58.8)	1	NA	NA	NA	NA

*NA*, not applicable for that analysis

### *G. duodenalis* typing results

Out of a total of 501 *G. duodenalis* PCR positive samples, 281 (56%) samples could be typed of which 132 and 149 samples were from children with diarrhea and children without diarrhea respectively. Assemblage B was found in 89% of samples followed by assemblage A (8%) and 3% was a mixed infection of assemblage A+B. Chi-square test was used to compare the differences in proportion of *Giardia* assemblage types in cases and control; results in Table 4 show that there were no significant differences (*P*=0.48) of the assemblage types between cases and control samples. We further assessed distribution of assemblages in two different age groups i.e., children aged <24 months (*n*=165) and children above 24 months (*n*=116). Out of 165 samples from children aged <24 months, 147 (89%) had assemblage B, while 15 (9%) and 3 (2%) had assemblage A and mixed assemblage A+B respectively. From 116 samples of children aged >24 months, 104 samples (90%) had assemblage B, 7 (6%) had assemblage A, and 5 (4%) had mixed assemblage A+B. There were no significant age differences in *G. duodenalis* assemblage distributions.

**Table 4:** Distribution of *Giardia* assemblage type among children with diarrhea and without diarrhea

Diarrhea status	Assemblage A	Assemblage B	Assemblage A+B	Total
Children with diarrhea	13 (10%)	115 (87%)	4 (3%)	132
Children without diarrhea	9 (8%)	136 (91%)	4 (3%)	149
Total	22 (8%)	251 (89%)	8 (3%)	281

## Discussion

This study is one of the few studies assessing the prevalence, assemblage, and risk factors of *Giardia* infection among pediatric population in Jimma Ethiopia. Additionally, to the best of our knowledge, this is one of the first studies to use assemblage-specific PCR and employing a case-control design in children under 5 years in Ethiopia.

Based on diagnostic real-time PCR, the overall prevalence of *G. duodenalis* among participants in this case-control study was 40%. In earlier studies within the country, some indicated a lower prevalence of 10–18% (Birrie and Erko 2017; Hajare et al. 2022; Wegayehu et al. 2016) while one study in North-west Ethiopia reported higher prevalence of 55% (de Lucio et al. 2016) than the current study. Comparing our findings with different African countries, there are differences in prevalence reported where some countries reported low *G. duodenalis* prevalence; Kenya 4.5% (Mbae et al. 2016), Tanzania 4.6% (Tellevik et al. 2015), Ghana 5.6% (Anim-Baidoo et al. 2016), Egypt 24.4% (Ahmad et al. 2020), and Mozambique 27.4% (Messa Jr et al. 2021). Other countries such as Rwanda (Ignatius et al. 2012) and Uganda (Al-Shehri et al. 2019) reported high *Giardia* prevalence of 60.1% and 87%, respectively. The marked variation in *G. duodenalis* prevalence is likely related to the varying sensitivity of the diagnostic methods used across studies. Studies which used PCR, generally considered a more sensitive method (Stensvold and Nielsen 2012), tend to report higher prevalence (de Lucio et al. 2016; Ignatius et al. 2012; Al-Shehri et al. 2019) compared to studies using a conventional microscopy (Mbae et al. 2016; Ahmad et al. 2020; Messa Jr et al. 2021). Secondly, the age of the study population matters, as shown in this study *G. duodenalis* detection increased with age. Studies which included older children like Rwanda (Ignatius et al. 2012) and other parts of Ethiopia (de Lucio et al. 2016) reported high *G. duodenalis* prevalence. Thirdly, geographical variation and socio-economic factors, such as access to safe drinking water, sanitation, and hygiene practices, may also be the cause of variation in *G. duodenalis* prevalence (Nundy et al. 2011). Some studies, e.g., in Tanzania (Tellevik et al. 2015) and Ethiopia (Wegayehu et al. 2016) reported low prevalence of *G. duodenalis* of 4.6% and 16.8% despite using PCR.

We found high prevalence of *G. duodenalis* among participants in this case-control study without significant differences even after removing from the analysis children with a history of diarrhea in the past month. Children without diarrhea with *G. duodenalis* may serve as parasite reservoirs. Several studies in developing countries reported higher prevalence of *G. duodenalis* in controls than in cases with acute diarrhea (Tellevik et al. 2015; Anim-Baidoo et al. 2016; Ahmad et al. 2020; Messa Jr et al. 2021; Ignatius et al. 2012; Al-Shehri et al. 2019; Becker et al. 2015; Muhsen and Levine 2012) and the parasite is overlooked in from global burden estimations of diarrheal diseases (GBD 2013 Mortality and Causes of Death Collaborators 2015). The frequent detection of *Giardia* in both case and control groups serves as a compelling indication that waterborne pathogens are commonly transmitted among the studied age groups. Consequently, future research endeavors should explore detection of additional pathogens such as bacterial and viruses to precisely identify the specific etiological factors contributing to diarrhea within this study cohort.

*G. duodenalis* infection was significantly associated with increasing age. Age-related increase in prevalence of infection has also been observed in other studies (Mbae et al. 2016; Tellevik et al. 2015; Ignatius et al. 2012). Children without diarrhea with history of diarrhea for the last one month before the study had increased risk of *Giardia* detection from stool. This could be due to prolonged shedding of *G. duodenalis* cyst in stool from the previous diarrhea episode or asymptomatic giardiasis. No other demographic or clinical characteristic assessed in our study such as sex, acute malnutrition, and keeping animals in the house were associated with *G. duodenalis.*

One of the limitations of the present study was that only 50% of the samples could be typed. Based on the assemblage-specific real-time PCR employed, *G. duodenalis* assemblage B was the predominant subtype with a prevalence of 89%. Similarly, assemblage B was reported in higher frequency than assemblage A in previous studies conducted in Ethiopia (de Lucio et al. 2016; Wegayehu et al. 2016) and other African countries (Tellevik et al. 2015; Messa Jr et al. 2021; Ignatius et al. 2012; Al-Shehri et al. 2019). On the other hand, assemblage A was also reported higher in some other studies in Ethiopia and other countries (Gelanew et al. 2007; Hajare et al. 2022; Ahmad et al. 2020). The variation in the predominance of *G. duodenalis* assemblage may partly be explained by either the genetic diversity of *G. duodenalis* circulating in different geographical areas or the difference in the dynamics of transmission. There are controversial reports on the role of *G. duodenalis* assemblages and clinical symptoms (Haque et al. 2009; Haque et al. 2005; Molina et al. 2011; Read et al. 2002). In this study, we did not find significant differences in *G. duodenalis* assemblages between children with and without diarrhea. However, studies from Rwanda, Australia, and Bangladesh have shown that *G. duodenalis* assemblage A was more likely to be found in symptomatic diarrheic children (Ignatius et al. 2012; Haque et al. 2009; Haque et al. 2005; Read et al. 2002), while in Argentina children presented with abdominal pain were more likely to be found with *G. duodenalis* assemblage B (Molina et al. 2011). Further molecular studies are needed to clarify the association between *G. duodenalis* assemblage and diarrhea symptoms. In the present study mixed infection of *G. duodenalis* assemblage A and B was found in 3 percent of cases and has been reported previously in Ethiopia and other countries (Hijjawi et al. 2022). The occurrence of mixed infections by several assemblages of *G. duodenalis* suggests the intricate circulation of the parasite within the environment and study participants may have been exposed to multiple sources of infection (Tamura et al. 2011). To comprehensively understand this, further studies are needed to characterize the origins of contamination and gain insight into varying contributions of anthroponotic, zoonotic, and environmental modes of transmission. Furthermore, we did not find any correlation between *G. duodenalis* assemblages and other clinical or socio-demographic parameters. This could be due to the strong dominance of assemblage B in our sample set which resulted in low power to assess correlations.

Higher risk of *G. duodenalis* in rural areas, has also been reported in other studies (Samie et al. 2020) and is likely caused by individuals, especially children, being in closer contact with natural sources of water and soil, thereby increasing risk of transmission (Addy et al. 2004). Increased in *G. duodenalis* infection prevalence during the rainy season was observed in studies conducted in Zambia (Siwila et al. 2011), Southwest London (Breathnach et al. 2010), and Canada (Brunn et al. 2019). In the present study, no clear seasonal pattern emerged.

## Conclusion

Using PCR method, we report a prevalence of 41% of *G. duodenalis* in this study from Jimma Ethiopia. Assemblage B was the predominant type causing giardiasis in this region. *Giardia* prevalence increased with age. The occurrence of diarrhea symptoms was not associated with *G. duodenalis* infection, nor with *Giardia* assemblage.

## Data Availability

All data generated or analysed during this study are included in this published article.
